# Temperature integrity and exposure of vaccines to suboptimal temperatures in cold chain devices at different levels in three states of India

**DOI:** 10.1186/s40794-020-00109-z

**Published:** 2020-06-03

**Authors:** Manoja Kumar Das, Narendra Kumar Arora, Thomas Mathew, Bhadresh Vyas, Salam Kabita Devi, Abhishek Yadav

**Affiliations:** 1 The INCLEN Trust International, F1/5, Okhla Industrial Area, Phase 1, New Delhi, 110020 India; 2 Department of Community Medicine, Government Medical College, Thiruvananthapuram, Kerala 695011 India; 3 Department of Pediatrics, M P Shah Medical College, Jamnagar, Gujarat 361008 India

**Keywords:** Cold chain, Vaccines, Temperature maintenance, Temperature excursion, Freezing, Datalogger

## Abstract

**Objective:**

To document the temperature integrity at the vaccine storage devices at various levels in three states of India.

**Methods:**

A total of 213 health facilities including 196 facilities (district and sub-district levels) from 27 select districts and 17 division or state level vaccine stores in three states were included. At these facilities, temperature in 223 vaccine storage devices was recorded for at least 7 consecutive days using electronic temperature datalogger.

**Results:**

During the observation period, overall the vaccines were exposed to temperature < 0 °C for 14.8% of the storage time with 8.6, 6.7 and 18% at state/division, district and sub-district vaccine stores, respectively. The vaccines were also exposed to temperature > 8 °C for 6.6% of the storage time including 1.3, 13 and 5.1% at state/division, district and sub-district vaccine stores, respectively. Continuous episodes of temperature deviation for 45 min or longer to < 0 °C and > 8 °C was observed in 7.2 and 6.4% of the observation period, respectively. These temperature deviations were not captured by the routine temperature monitoring practice.

**Conclusion:**

The vaccines were exposed to freezing temperature for a considerable period at all level stores, which was more than the exposure to higher temperature. To ensure vaccine potency and immunogenicity, stringent temperature integrity maintenance is needed at all levels.

## Background

Vaccination is one of the most cost-effective public health interventions in twenty-first century [1]. Vaccines are averting millions of deaths and sequelae due to diseases globally and have achieved smallpox eradication, poliomyelitis control and now aiming for measles eradication. Vaccines are temperature sensitive products and requires storage within narrow temperature range (usually 2-8 °C) during storage and transfers to preserve potency [2]. Some vaccines like oral polio vaccine (OPV) and rotavirus vaccine may be stored at -20 °C at bulk stores [3]. All vaccines are usually damaged by cumulative exposure to temperatures above + 8 °C. Vaccine vial monitor (VVM) containing a heat-sensitive material indicate the cumulative heat exposure and guide use of the vaccine. Exposure to freezing temperatures (< 0 °C) is equally damaging for the freeze-sensitive vaccines like diphtheria-pertussis-tetanus (DPT), liquid pentavalent vaccine (LPV), tetanus toxoid (TT), hepatitis A and B, human papillomavirus (HPV), meningitis C, pneumococcal conjugate vaccine (PCV), cholera, influenza, haemophilus influenza b (Hib), and inactivated poliovirus (IPV) making them inactive [4]. Apart from losing potency, administration of frozen vaccines may cause some adverse events [3]. Detection of exposure to freezing is challenging, as there is no marker currently available and the shake test is not commonly practiced by cold chain handlers and vaccinators [5, 6]. For shake test, the vaccine vial is vigorously shaken and placed inverted on a flat surface and examined for the physical changes, and the extent of sedimentation after 30 min. The presence of granular forms or floccules when shaking, or the formation of a sediment at the bottom of the container within 30 min, with clear liquid above, suggest that the vaccine has been frozen [7, 8]. The Universal Immunization Programme (UIP) of India targets about 26 million infants, 100 million children aged 1–5 years and 30 million pregnant women annually [3]. The UIP currently delivers vaccines against 13 vaccine-preventable diseases to children and pregnant women [3]. The cold chain in India is a five-tier storage system with about 26,388 cold chain points or vaccine stores (VSs), including the government medical supply depots (GMSDs) (*n* = 4), state VSs (*n* = 53), divisional VSs (*n* = 110), district VSs (*n* = 666) and the sub-district VSs (*n* = 25,555) at community health centers (CHCs) and primary health centers (PHCs) [9]. At the GMSDs, the vaccines are stored in cold rooms, walk-in coolers (WICs) and walk-in freezers (WIFs). At state and division VSs, the vaccines are usually stores in WICs, ice-lined refrigerators (ILRs) and WIFs. At district and sub-district (CHCs and PHCs) VSs, the vaccines are stored in ILRs. The cold rooms, WICs and ILRs are expected to maintain the optimum temperature (+ 2 to + 8 °C) for vaccine storage. The WIFs and deep freezers (DFs) are expected to maintain the optimum temperature (− 15 to -25 °C) for long-term vaccine storage. The vaccines are transferred in cold boxes of various sizes (between the VSs) and vaccine carriers (to outreach sessions) with icepacks to maintain temperature in + 2 to + 8 °C range. The vaccines and related supplies are handled by cold chain handlers at the cold chain points. The cold chain equipment are maintained by cold chain technicians.

Several studies from India reported exposure of vaccines to high temperatures [10–12]. The literature reported exposure of vaccines to freezing temperatures at vaccine stores for 10–33.7% in developing countries and 6.4–22.5% in developed countries [13, 14]. In India, a study across ten states informed about frequent exposure of the vaccines to freezing temperature at state compared to district, division and sub-district VSs [15]. This study was conducted to assess the available cold chain space and vaccine temperature integrity in three states of India for new vaccine introduction. This paper presents the data related to the temperature integrity at the cold chain points.

## Methods

### Study design and setting

**Fig. 1:**
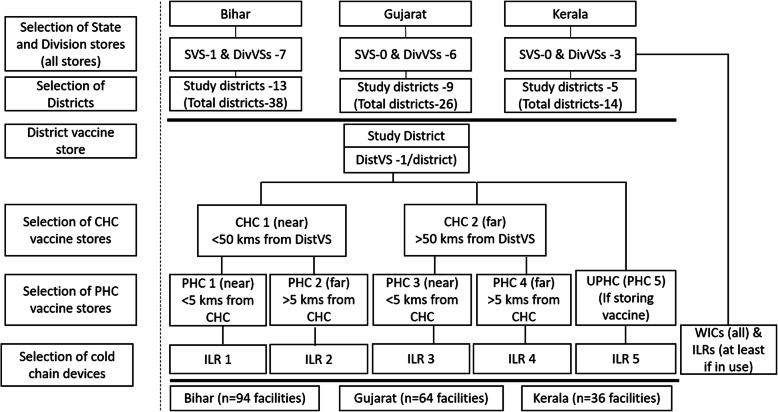
Process of selection of facilities and cold chain devices in the states and study districts

This cross-sectional study was undertaken in three states of India: Bihar, Gujarat and Kerala during 2014–15. The states were purposively selected representing three different levels of routine immunization coverage (lowest, Bihar 49%, moderate, Gujarat 56.6% and highest, Kerala 81.5% according to Coverage Evaluation Survey 2009), governance and geography [16]. In each state one-third districts were included. Thus, in Bihar, 13 of 38 districts, in Gujarat, 9 of 26 districts and in Kerala, 5 of 14 districts were randomly selected, ensuring at least one district per division. In each selected district, seven VSs were selected through a multistage process based on distance from the district VS, as shown in Fig. 1. The state and district immunization program teams were consulted for selection of the districts and facilities in the districts. Additionally, in each state, all the division and state VSs were included and the WIC and ILR (used for vaccine storage) were selected for temperature monitoring.

### Temperature recording at stores

The study followed WHO protocol for temperature monitoring in the vaccine cold chain [17]. We used LogTag-Trix8 data loggers (range -40 °C to + 85 °C; precision ±0.5 °C) to record the temperatures (LogTag Recorders, Auckland, New Zealand) every 15 min [18]. The data logger battery and memory could record 8000 readings continuously. Temperature monitoring units (*n* = 145) containing one datalogger and two DPT vials (shake test negative) were placed inside the WICs, ILRs and others devices storing the vaccines at these facilities for at least 1 week. The research staff installed and removed the dataloggers for each device. For each cold chain device, the datalogger ID, installation and removal time, position of the data logger, thermometer availability, ice deposit on walls, gasket and door seal status at installation and removal were documented. The temperature chart recorded by cold chain handler (for the 1 week observation period and past 3 months) and verifications by the medical officer in-charge (from the registers) were documented at the time of logtag removal. One freeze sensitive vaccine (DPT/TT/HBV/LPV) from each box stored in the ILR and each rack of WIC (2–3 per ILR/WIC) were checked for freezing by shake test. All the dataloggers were transported to central coordinating office along with the filled data forms. The readings from the data loggers were downloaded using the dedicated software and exported into excel, which contained date and time stamp for each temperature record.

### Data analysis

The first and last data points for the data loggers were excluded, as these might be representing the environmental temperature at placement and removal time. The exposure of vaccines to temperatures < 0 °C, ≥0 °C to < 2 °C, ≥2 °C to ≤8 °C and > 8 °C as proportions of observation period were estimated. Lower post-immunization IgG responses to the DTP vaccines (both acellular and whole cell pertussis antigens) in murine model has been reported on exposure to freezing at − 3 °C for 24 h [19]. Thus, the exposure to temperatures < 0 °C were further segregated to <− 3 °C and ≥ − 3 °C to 0 °C to document the risk of freezing. It was observed that after opening of the ILRs (with the logtags inside), the temperature increased and reached to preopening range after 15–30 min. Thus continuous episodes (periods with more than two consecutive readings by a data logger in the same range, for ≥45 min) of temperature excursion (remaining below 0 °C or above 8 °C), segregated for different durations (<1 h, > 1–9 h and ≥ 10 h) were also calculated. The duration of temperature maintenance in the devices were estimated for different types of devices (WICs, ILRs and others) and levels of vaccine stores (state, division, district and sub-district). The data for state and division VSs were pooled, as there was no state store in Gujarat and Kerala. Descriptive statistics measures were calculated using Microsoft Excel and STATA version 12 (StataCorp LLC, Texas, USA).

### Approvals

Administrative approvals from the appropriate national and state health authorities and ethical approval from INCLEN institute ethics committee (protocol no IIEC-006) were obtained.

## Results

The temperature recordings from 196 facilities and 218 devices were received. The recordings from 5 ILRs (from 3 PHCs and 2 District VSs) were less than 7 complete days and excluded from analysis. The temperature records for 59,039 h from 213 devices from 191 facilities were analysed. Table 1 shows the distribution of the types of facilities and devices. At 4 PHCs, non-ILR devices (domestic refrigerator, *n* = 1 and cold boxes, *n* = 3) were used for vaccine storage, due to temporary breakdown of ILRs.

**Table 1:** Description of vaccine stores and cold chain devices included for temperature monitoring

Level/Devices		States			Total
		Bihar	Gujarat	Kerala	
Level of stores	State VS	1	0	0	1
	Division VS	7	6	3	16
	District VS	10	9	5	24
	CHC/PHC	76	49	30	155
	Total	94	64	36	196
Type of cold chain devices	WIC	10	6	4	20
	ILR	95^a^	61	38	189
	Refrigerator	1	0	0	1
	Cold box	3	0	0	3
	Total	109	67	42	218

*VS* Vaccine store, *CHC* Community health centre, *PHC* Primary health centre, *ILR* Ice lined refrigerator, *WIC* Walk-in cooler, *Refrigerator* Domestic refrigerator

^a^Recordings from 5 ILRs (from 3 PHCs and 2 District vaccine stores) were less than 7 complete days and excluded from analysis

### Temperature maintenance at vaccine stores

As shown in Table 2, during the observation period, 75.4% (95% CI: 60.8, 89.1%) of the state and division VSs; 71.1% (95% CI: 55.6, 83.1%) of the district VSs and 56.7% (95% CI: 50.2, 63.1%) of the sub-district (CHCs/PHCs) VSs maintained temperature in the desired range. The temperature dipped to sub-zero range for 8.5% (95% CI: 1, 17.1%), 7.4% (95% CI: 6.3, 8.4%) and 17.7% (95% CI: 11.9, 22%) of the observation period at the state/division, district and sub-district VSs respectively. The temperature also crossed above 8 °C in 5.2% (95% CI: 1, 11.3%), 10.1% (95% CI: 3.8, 16.3%) and 5.4% (95% CI: 5.1, 5.6%) of the observation period at the state/division, district and sub-district VSs respectively. The temperature fluctuated widely, from -33 °C to + 35 °C in the devices. The temperature deviation to both extremes (< 0 °C and > 8 °C) was observed at 19.7% of the devices (state/division- 15%, district- 12.9% and sub-district- 21.9%). The vaccines were exposed to sub-zero temperatures for longer period at the state/division and sub-district VSs and pooled for all levels, than exposure to > 8 °C. The vaccines spent sizable proportions of time at <− 3 °C (freezing temperature for many freeze-sensitive vaccines) at all levels of facilities (2.9 to 7.2%) and in all types of devices (0.4 to 6.7%), more in Bihar (8.5%). The vaccines stored in non-standard cold chain devices (domestic refrigerators/cold boxes) were exposed to > 8 °C for 55% of the observation period.

**Table 2:** Temperature integrity in cold chain devices according to type of vaccine stores

Levels/ Devices	State	Duration^a^ (hours)	% of time spent					Average temp (°C)	Temperature range (°C) (min, max)
			<− 3 °C	-3 °C to 0 °C	0 °C to 2 °C	2 °C to 8 °C	> 8 °C		
According to levels of stores									
State and Divisions	Bihar	4107.5	4.5	8.6	12.8	66.6	7.4	2.7	−16.1, 26.1
	Gujarat	1395	0.1	0.3	11.2	86.2	1.9	4.8	−7.2, 19.7
	Kerala	860.5	0	0	0	99.6	0.4	4.6	2.1, 33.2
	Total	6363	2.9	5.6	10.7	75.4	5.2	3.8	−16.1, 33.2
District	Bihar	5163.7	6.4	6.5	16.8	44.7	25.5	2.6	−22.9, 23.9
	Gujarat	3228.7	0	0.1	2	97.4	0.4	4.8	−1.2, 25.3
	Kerala	1914.5	0	1	1.1	97.9	0	3.7	−1.5, 12.8
	Total	10,307	3.2	3.5	9.3	71.1	12.9	3.6	−22.9, 25.3
Sub-district	Bihar	23,728.2	9.6	10.1	14.5	58	7.8	2.4	−33.0, 34.9
	Gujarat	12,115.2	6.3	13.7	25.1	53.4	1.5	2.0	−14.1, 33.3
	Kerala	6526.2	0	6.5	36.8	53.9	2.8	2.8	−2.6, 34.6
	Total	42,369.7	7.2	10.5	21	56	5.2	2.4	− 33.0, 34.9
According to type of devices									
WIC	Bihar	3483.2	0.5	8.2	10.6	72	8.6	4.4	−4.6, 26.1
	Gujarat	1036.7	0.1	0.4	15.1	81.5	2.5	4.6	−7.2, 19.7
	Kerala	718.5	0	0.	0	99.5	0.5	4.3	2.1, 33.2
	Total	5238.5	0.4	5.5	10.1	77.6	6.3	4.4	−7.2, 33.2
ILR	Bihar	28,452.2	9.7	9.8	14.9	56.4	9.1	1.9	−33.0, 34.6
	Gujarat	15,702.2	4.8	10.6	19.8	63.5	1.3	2.7	−14.1, 33.3
	Kerala	8582.7	0	5.1	28.2	64.5	2.1	3.0	−2.6, 33.2
	Total	52,737.2	6.7	9.3	18.5	59.9	5.6	2.4	−33.0, 34.6
Other devices^b^	Bihar	1064	0	0.3	21.9	22.1	55.5	9	−2.3, 24.6
	Total	1064	0	0.3	21.9	22.1	55.5	9	−2.3, 24.6
Pooled for all levels and all types of devices									
Pooled all levels	Bihar	32,999.5	8.5	9.3	14.7	57	10.5	2.4	−33.0, 34.9
	Gujarat	16,739	4.5	9.9	19.5	64.6	1.4	2.8	−14.1, 33.3
	Kerala	9301.2	0	4.7	26	67.2	2	3.2	−2.61, 34.6
	Total	59,039.7	6	8.8	17.8	60.8	6.6	2.7	−33.0, 34.9

*WIC* Walk-in cooler, *ILR* Ice lined refrigerator

^a^Duration of temperature recording by data loggers in the cold chain devices in hours

^b^ Other devices (domestic refrigerator and cold boxes) were used for vaccine storage in Bihar only at the time of the study

### Continuous episodes of temperature excursion

The continuous temperature excursion episodes were observed at all levels of VSs, as reflected in Table 3. The pooled continuous episodes (≥45 min) constituted to 14.8% of the temperature excursion to the sub-zero temperature ranges. The proportion of continuous excursion episodes were higher in the devices at all levels in Bihar; 13.1% at state/division, 12.9% at districts and 19.7% at sub-district VSs, which were higher than Gujarat and Kerala. The continuous excursion episodes to sub-zero temperature at the sub-district VSs in Gujarat were also high (19.9%).

**Table 3:** Continuous episodes of temperature excursion below 0 °C in cold chain devices

		Total observation period^a^	Exposure to temperature < 0 °C				
			Total duration^a^ (hours)	Total continuous episodes duration^a^ (hours)	Duration of continuous episodes^b^		
					≤1 h	> 1–9 h	≥10 h
State and Division	Bihar	4107.5	540	28.9	12.7	14	2.2
	Gujarat	1395	5.75	0.7	0.2	0.5	0
	Kerala	860.5	0	0	0	0	0
	Total	6363	545.7	29.7	13	14.5	2.2
District	Bihar	5163.7	666.7	5.9	0.7	2.7	2.5
	Gujarat	3228.7	4.5	0.7	0	0.7	0
	Kerala	1914.5	19	1	0	1	0
	Total	10,307	690.2	7.7	0.7	4.5	2.5
Sub-district	Bihar	23,728.2	4663.2	41.9	5.2	19	17.7
	Gujarat	12,115.2	2415.7	62.9	29.2	26.2	7.5
	Kerala	6526.2	421.2	14.9	3	10.2	1.7
	Total	42,369.7	7500.2	120	37.5	55.5	27
WICs	Bihar	3483.2	303.2	22.2	12.2	8	2
	Gujarat	1036.7	5.75	0.7	0.2	0.5	0
	Kerala	718.5	0	0	0	0	0
	Total	5238.5	309	23	12.5	8.5	2
ILRs	Bihar	28,452.2	5563.2	53.7	5.7	27.5	20.5
	Gujarat	15,702.2	2420.2	63.7	29.2	27	7.5
	Kerala	8582.7	440.2	16.4	3	11.7	1.7
	Total	52,737.2	8423.7	133.4	38	65.7	29.7
Other devices^c^	Bihar	1064	3.5	0.9	0.7	0.2	0
	Total	1064	3.5	0.9	0.7	0.2	0
Pooled all levels	Bihar	32,999.5	5870	76.9	18.7	35.7	22.5
	Gujarat	16,739	2426	64.5	29.5	27.5	7.5
	Kerala	9301.2	440.2	16.4	3	11.7	1.7
	Total	59,039.7	8736.2	157.7	51.5	74.5	31.7

*WIC* Walk-in cooler, *ILR* Ice lined refrigerator

^a^ Duration in hours

^b^ A continuous episode below 0 °C was counted when readings were continuously below 0 °C for at least 45 min or more

^c^ Other devices (domestic refrigerator and cold boxes) were used for vaccine storage in Bihar only at the time of the study

As shown in Table 4, the pooled continuous excursion episodes constituted 6.6% of the temperature excursion duration to > 8 °C range. The continuous excursion episodes to > 8 °C were higher in Bihar (6.6%) than Gujarat and Kerala. The continuous excursion episodes in Bihar were higher at district (25.5%) followed by state/division (7.4%) and sub-district level VSs (7.8%).

**Table 4:** Continuous episodes of temperature excursion above 8 °C in cold chain devices

		Total observation period^a^ (hours)	Exposure to temperature > 8 °C				
			Total duration^a^ (hours)	Total continuous episodes duration^a^ (hours)	Duration of continuous episodes^b^		
					≤1 h	> 1–9 h	≥10 h
State and Divisions	Bihar	4107.5	302.7	14.5	4.25	8	2.25
	Gujarat	1395	26	7	7	0	0
	Kerala	860.5	3.5	0.2	0	0.25	0
	Total	6363	332.2	21.7	11.25	8.25	2.25
District	Bihar	5163.7	1318.7	2.2	0	0.5	1.75
	Gujarat	3228.7	14.5	0.5	0.25	0	0.25
	Kerala	1914.5	0.5	0.2	0.25	0	0
	Total	10,307	1333.7	3	0.5	0.5	2
Sub-district	Bihar	23,728.2	1846.2	28.5	8.75	11.75	8
	Gujarat	12,115.2	186	6.5	4	1.2	1.2
	Kerala	6526.2	182.7	2.2	0.5	0	1.7
	Total	42,369.7	2215	38.2	13.5	12.7	12
WICs	Bihar	3483.2	299.2	13.7	3.7	8	2
	Gujarat	1036.7	26	7	7	0	0
	Kerala	718.5	3.5	0.2	0	0.2	0
	Total	5238.5	328.7	21	10.7	8.2	2
ILRs	Bihar	28,452.2	2578	27.2	9	11.2	7.5
	Gujarat	15,702.2	200.5	7.5	4.5	1.2	1.5
	Kerala	8582.7	183.2	2.5	0.7	0	1.7
	Total	52,737.2	2961.7	37.2	14.2	12.5	10.7
Other devices^c^	Bihar	1064	590.5	4.75	0.7	0.7	3.2
	Total	1064	590.5	4.7	0.7	0.7	3.2
Pooled all levels	Bihar	32,999.5	3467.7	46.2	13.5	20	12.7
	Gujarat	16,739	226.5	14.5	11.5	1.2	1.5
	Kerala	9301.2	186.7	2.7	0.7	0.2	1.7
	Total	59,039.7	3881	63.2	25.7	21.5	16

*WIC* Walk-in cooler, *ILR* Ice lined refrigerator

^a^ Duration in hours

^b^ A continuous episode above 8 °C was counted when readings were continuously above 8 °C for at least 45 min or more

^c^ Other devices (domestic refrigerator and cold boxes) were used for vaccine storage in Bihar only at the time of the study

### Routine temperature monitoring practices

At the state/division VSs, 76% of the WICs (Bihar-63.6%, Gujarat-90% and Kerala-75%) and 71.4% of ILRs (Bihar-72.7%, Gujarat-80% and Kerala- 61.5%) had functional temperature monitors at the time of observation. The functional thermometers were available in 83.6% (Bihar-76.3%, Gujarat-74.6% and Kerala-100%) of ILRs at district VSs and 97% (Bihar-94.7%, Gujarat-100% and Kerala-96.4%) of ILRS at sub-district VSs. The temperature logbook was available at all state/division, 81.7% of the district (Bihar-81.8%, Gujarat-83.3% and Kerala-80%) and 84.5% of the sub-district VSs (Bihar-66.5%, Gujarat-92.6% and Kerala-94.3%). From the temperature logbooks, deviation in temperature (< 2 °C/> 8 °C) for the observation period (period of logtag installation) was observed in only 5.6% of devices (Bihar, *n* = 7; Gujarat, *n* = 4 and Kerala, *n* = 1). According to the logbooks, deviations in temperature to < 2 °C or > 8 °C were observed at 5.8% facilities (Bihar, n = 7; Gujarat, *n* = 3 and Kerala, n = 4) during 3 months prior to the survey. Weekly supervision of temperature logbook by the in-charge officer (bearing signature) was observed at 24.1% facilities and at least one supervision was done at 29.6% facilities within last 1 month. Gaskets were not fitting/damaged in 23.3% of the devices and ice layer > 5 mm was observed in 2% ILRs. The documentation for preventive maintenance of the devices were not available at most (85%) of the facilities, more at the district and sub-district level.

Frozen freeze sensitive vaccine vials were observed in 12 PHCs in Bihar (from 5 districts), 7 PHCs in Gujarat (from 3 districts) and 6 CHCs/PHCs in Kerala (from 4 districts). The study team didn’t check the all the vaccine vials in the ILRs/WICs for freezing or damage. No vaccine vial was discarded due to freezing or damage at any of the surveyed facilities (VSs) as per the store records.

## Discussion

Immunization is a critical component of the Government of India’s child survival strategy, elimination and control of vaccine preventable diseases (maternal and neonatal tetanus, poliomyelitis, measles, etc.) and achieving sustainable development goal of ending preventable deaths of newborn and under-five children by 2030. In 2017, India’s immunization budget was estimated to 1480 million USD which has almost doubled compared to 2012 (around 700 million USD). The share of vaccine cost has increased from 11% in 2012 to 38% in 2017 (around 560 million USD) with introduction of the new vaccines [20]. Maintenance of optimal cold-chain system across all levels is inevitable to ensure administration of good quality and potent vaccines to each child or woman in the country.

During the observation period, temperature excursion outside + 2 to + 8 °C range was documented for sizable proportion of storage times (24.6 to 43.9%) at all levels. Temperature excursions to both extremes of temperature range, < 0 °C and > 8 °C were observed at all levels. The vaccines were exposed to continuous episodes of temperature excursion, longer to < 0 °C range (14.8%) than to > 8 °C range (6.6%). The exposure to sub-zero temperature at the state/division vaccine stores was worrisome in view of the duration exposure, continuous episodes and bulk storage of vaccines for longer period. The reasons for higher temperature excursions observed in devices in one state (Bihar) were not clearly known.

Another study from India (10 states; 131 facilities) reported exposure of vaccines to > 8 °C for 8.3–14.7% (14.3, 13.2, 8.3 and 14.7% for state, divisional, district and sub-district vaccine stores respectively) and to below 0 °C for 0.2–10.5% (1.5, 0.2, 0.6 and 10.5% for state, divisional, district and sub-district vaccine stores respectively). While state stores in eight states documented excursion to > 8 °C temperature (0–83.3%), only one state (Tamil Nadu) experienced excursion to sub-zero temperature (10.7%). The exposure to sub-zero temperature (10.5%) was comparable that for > 8 °C (14.7%) at sub-district stores. Continuous episodes constituted majority of the temperature deviations; about 85 and 94% of the exposure to temperatures of < 0 °C and > 8 °C respectively [15]. The two states (Bihar and Gujarat) were common with the current study. At state/division level stores, the vaccines were exposed to > 8 °C for 16.6 and 0.5% of observation periods respectively. In Bihar, at district and sub-district level stores, the vaccines were exposed to > 8 °C for 49 and 39.5% of observation periods respectively. No temperature excursion was observed at district levels stores in Gujarat. The vaccines were exposed to sub-zero temperature for 2.6 and 11.6% of observation period in Bihar and Gujarat respectively. Several other studies also reported temperature integrity challenges at vaccine stores and outreach levels in India [10–12, 21, 22].

Review of articles (14 studies; during 1985–2006) summarized that at storage level, 21.9% (95% CI: 10.3–33.6%) refrigerators in developing countries were exposed to freezing temperature compared to 13.5% (95% CI: 6.4–20.7%) in developed countries [13, 23–25]. A follow up review of articles (18 studies including 8 from developing countries and 10 from developed countries; during 2006–2014) documented that at storage level, 37.1% (SD: 29.7) of the refrigerators in developing countries and 33.3% (SD: 22.5) in developed countries [14, 15, 26–28]. These two reviews indicate persistence of the temperature excursion at the vaccine storage points, despite the possible variations in study methodologies and rigors. The practices of manual temperature monitoring were not able to capture these temperature excursions. According to reports, the practice of detection of freezing by shake test for freeze-sensitive vaccines had been suboptimal, both at vaccine stores (22.4%) and vaccination sites (8.6%) [6, 21, 29].

The stability and potency of vaccines are generally temperature-sensitive. Effect of vaccine exposure to higher temperature is usually cumulative and can alter the protein structure and/or chemical stability leading to loss of potency. VVM indicates the cumulative exposure to heat (duration * degree of heat). Exposure of freeze-sensitive vaccines to sub-zero temperatures cause dissociation of antigen from adjuvants, conglomeration of the aluminium salt adjuvants, and the granule size increase with repeated freeze-thaw cycles [8, 30]. Administration of frozen DPT and TT vaccines are more likely to cause local reactions [30, 31]. Exposure of DPT vaccines to freeze-thaw cycles have demonstrated progressive drop in potency with number of cycles [32]. Instances of vaccine failure and suboptimal population immunity with vaccination with the frozen freeze-sensitive less potent vaccines has been reported for hepatitis B in Mongolia and pertussis in USA [33, 34].

This study documented the temperature integrity and occurrence of excursions at the vaccine stores of all levels in the three states. These temperature excursions were not captured by the routine manual temperature monitoring. The issues emerged that require attention for safe storage of the vaccines were: lack of routine maintenance practices including defrosting, thermostat setting according to ambient temperature, irregular power supply, shortages of trained and dedicated personnel, calibration of thermometers and periodic supervision to ensure cold chain maintenance, especially at the sub-district levels. It may be important to assess the impact of temperature deviations and continuous episodes on freezing and damage of the vaccine vials in the WICs/ILRs in future studies.

The recent efforts of Government of India on Electronic Vaccine Intelligence Network (eVIN) is a step towards ensuring vaccine temperature integrity at cold chain stores [35]. The eVIN system is being expanded in a phased manner in India. Efforts towards training of the cold chain handlers are also being undertaken. Also till eVIN is implemented universally, availability of functional thermometer in all cold chain devices and appropriate documentation are to be ensured. Apart from the stringent temperature monitoring, capacity building of the dedicated cold chain handlers and technicians for routine device maintenance, ensuring appropriate power supply, suitable voltage stabilizers and regular supervision and monitoring are needed to ensure cold chain integrity, especially at the district and sub-district levels.

Our study has certain limitations. We did not use additional freeze alert to indicate freezing. Shake test was not done for all the eligible vaccine vials. Shake test was done for the vaccine vials from the devices only once, at the time of LogTag removal, thus might not inform about the past freezing exposures. The potency of vaccines exposed to either higher or lower temperatures was not tested.

## Conclusions

The study documented that vaccines were exposed to freezing temperature for a considerable period at all level stores and the exposure duration was more than that to the higher temperature. These sub-zero temperature excursions were not captured by the routine manual temperature monitoring and indicated by any VVM like indicator. Exposure of the freeze-sensitive vaccines to sub-zero temperature can affect the vaccine potency and reduce effectiveness. To ensure vaccine potency and immunogenicity, stringent temperature integrity maintenance is needed at all levels. There is need for refresher capacity building of cold chain handlers and store in-charges in preventive device maintenance, ensuring periodic physical audits and systematic supportive supervision.

## Data Availability

The data used to support the findings of this study can be obtained from the corresponding author on request.

## Abbreviations

CHC: Community health center
CI: Confidence interval
DF: Deep freezer
DPT: Diphtheria-pertussis-tetanus
eVIN: Electronic Vaccine Intelligence Network
GMSD: Government medical supply depots
HB: Hepatitis B
Hib: Haemophilus influenza b
HPV: Human papillomavirus
ILR: Ice-lined refrigerator
IPV: Inactivated poliovirus
LMIC: Low and middle income countries
LPV: Liquid pentavalent vaccine
OPV: Oral poliovirus vaccine
PCV: Pneumococcal conjugate vaccine
PHC: Primary health center
SD: Standard deviation
TT: Tetanus toxoid
UIP: Universal immunization program
USD: United States Dollar
VVM: Vaccine vial monitor
WHO: World health organization
WIC: Walk-in cooler
WIF: Walk-in freezer
